# A multicentre phase II study of carboplatin plus pegylated liposomal doxorubicin as first-line chemotherapy for patients with advanced or recurrent endometrial carcinoma: the END-1 study of the MITO (Multicentre Italian Trials in Ovarian Cancer and Gynecologic Malignancies) group

**DOI:** 10.1038/sj.bjc.6603787

**Published:** 2007-05-08

**Authors:** S Pignata, G Scambia, C Pisano, E Breda, M Di Maio, S Greggi, G Ferrandina, D Lorusso, V Zagonel, A Febbraro, N Riva, V De Rosa, C Gallo, F Perrone

**Affiliations:** 1 Instituto Nazionale Tumori, UOC Oncologia Medica B, Napoli, Italy; 2 Università Cattolica del Sacro Cuore, UOC Ginecologia Oncologica, Roma, Italy; 3 Ospedale S. Giovanni Calabita- Fatebenefratelli, UOC Oncologia Medica, Roma, Italy; 4 Istituto Nazionale Tumori , UOC Sperimentazioni cliniche, Napoli, Italy; 5 Istituto Nazionale Tumori , UOC Ginecologia Oncologica, Napoli, Italy; 6 Università Cattolica del Sacro Cuore, UOC Ginecologia Oncologica, Campobasso, Italy; 7 Ospedale FatebeneFratelli, UOC Oncologia Medica, Benevento, Italy; 8 Azienda Ospedaliera Pierantoni, UOC Oncologia Medica, Forlì, Italy; 9 Istituto Nazionale Tumori, UOC Radiologia, Napoli, Italy; 10 Seconda Università di Napoli, Cattedra di Biostatistica, Napoli, Italy

**Keywords:** endometrial cancer, advanced, chemotherapy

## Abstract

Anthracyclines and platinum derivates are active drugs for advanced endometrial carcinoma (AEC), but new schedules with higher efficacy and better tolerability are needed. A phase II study was conducted to describe activity and tolerability of carboplatin (C)+pegylated liposomal doxorubicin (PLD) in patients with AEC. Patients with chemonaive AEC, PS ⩽2, aged <75 years, with at least one measurable lesion were eligible. Treatment was C (area under curve 5)+PLD (40 mg m^−2^) on day 1 every 4 weeks, up to six cycles. Forty-two patients were needed in a single-stage design, with at least 13 objective responses to define the treatment active. Forty-two patients were enrolled. Median age was 64 years (31–74). A total of 64% of patients were recurrent while 36% were advanced. Three complete (7%) and 22 partial responses (52%) were observed, for an overall response rate of 59.5% (95% exact CI: 43.3–74.3). One death potentially related to treatment was recorded (death at home for unknown reasons after 6th cycle). Other relevant toxicities (% of patients) were grade 3/4 neutropaenia 33%/14%, febrile neutropaenia 5%, grade 3/4 thrombocytopaenia 17%/5%, grade 3/4 anaemia 31%/2%. Skin toxicity was mild: grade 1 14%, grade 2 10%, grade 3 5%. Hair loss: complete 5%, partial 12%. The combination of carboplatin and PLD shows good activity and favourable toxicity as first-line chemotherapy of patients with AEC, deserving further studies in this setting.

Endometrial carcinoma is the most common invasive malignancy of the female genital tract. Most patients with the disease present with early stage, have a good prognosis and can be cured with surgery. On the other hand, patients with advanced or recurrent disease have a worse prognosis, and results obtained with systemic therapy are far from being impressive.

Recent systematic reviews of randomised clinical trials show that there is a limited body of evidence available to help clinicians make decisions about the treatment of advanced or recurrent endometrial cancer (Carey *et al*, 2006; Polyzos *et al*, 2006). Doxorubicin, with or without cisplatin, has long been considered the standard regimen for treating advanced or recurrent endometrial carcinoma. Single-agent chemotherapy with doxorubicin has achieved response rates in the range of 17–25% (Thigpen *et al*, 1994). When cisplatin was combined with doxorubicin, two randomised trials demonstrated improved responses with the combination. Absolute response rates increased from 25 to 42% in one study (Thigpen *et al*, 2004) and from 17 to 43% in the other trial (Aapro *et al*, 2003). Unfortunately, neither trial showed improved survival. The only randomised trial showing a survival advantage evaluated the addition of paclitaxel to cisplatin plus doxorubicin, but the three-drug arm was definitely more toxic, requiring filgrastim support and causing a significant increase in thrombocytopaenia and neurotoxicity (Fleming *et al*, 2004).

New schedules with higher efficacy and better tolerability are strongly needed for the treatment of patients with advanced or recurrent endometrial carcinoma. The use of carboplatin instead of cisplatin has been suggested to improve tolerability with the same efficacy (Santin *et al*, 2004). Pegylated liposomal doxorubicin (PLD) is an interesting formulation of doxorubicin, in which the anthracycline is encapsulated in liposomes to obtain pharmacokinetic properties not available with conventional formulation of the drug: lower plasma concentration peak, lower clearance, smaller distribution volume, longer half-life, and higher area under curve (AUC), resulting in a different toxicity profile (Vaage *et al*, 1993; Sakakibara *et al*, 1996; Vaage *et al*, 1997; Safra *et al*, 2001). Moreover, the size of the liposomes allows selective accumulation in the tumour vascular bed following extravasation through the leaky tumour vasculature (Vaage *et al*, 1997). Furthermore, the special coating (pegylation) of the liposomes is associated with reduced clearance by the mononuclear phagocyte system, thus helping to maintain active drug concentrations for a long time (Vaage *et al*, 1993; Sakakibara *et al*, 1996; Vaage *et al*, 1997; Safra *et al*, 2001).

A prospective phase II study was conducted by the MITO (Multicentre Italian Trials in Ovarian Cancer and Gynecologic Malignancies) cooperative group to describe activity and tolerability of the combination of carboplatin plus PLD as first-line chemotherapy for patients with advanced or recurrent endometrial carcinoma. The doses of carboplatin AUC 5 and PLD 40 mg m^−2^ were chosen on the basis of the results of the only study on this combination published when the study was planned (Verschraegen *et al*, 2001).

## MATERIALS AND METHODS

### Eligibility criteria and baseline assessment

Patients with cytological or histological diagnosis of advanced or recurrent endometrial carcinoma, with indication for chemotherapy (stages III and IV, recurrent), age <75 years, an Eastern Cooperative Oncology Group (ECOG) performance status ⩽2, at least one measurable lesion and life expectancy of at least 3 months were eligible. Exclusion criteria were prior or concurrent malignant cancer (except for non-melanoma skin cancer and for *in situ* carcinoma of the uterine cervix, if adequately treated), previous chemotherapy treatment, inadequate bone marrow function (white blood cells <4000 mm^−3^ or platelets <100 000 mm^−3^); abnormal renal function (total serum creatinine level >1.25 the upper normal limit), abnormal liver function (sAST or sALT >1.25 the upper normal limit), heart disease (heart failure, heart attack in the previous 6 months, atrioventricular block of any degree, serious arrhythmia). The protocol was approved by the Ethics Committee of the Coordinating center (National Cancer Institute, Naples, Italy) and of each participating centre. Written informed consent was obtained from each enrolled patient, before study entry.

Baseline evaluation included a complete physical examination and history, complete blood count and biochemistry with Ca-125 determination, electrocardiogram, chest X-ray, abdomino-pelvic CT-scan or nuclear magnetic resonance, abdomino-pelvic ultrasonography, bone scan with skeletal X-ray as indicated, and other examination were at the Investigators discretion.

### Experimental treatment regimen, dose modifications, and delays

Patients were treated with carboplatin, AUC 5, intravenously (i.v.), and PLD, 40 mg m^−2^, i.v., both drugs given on day 1, every 28 days. Chemotherapy was administered for a maximum of six cycles. Carboplatin was dosed in accordance with the Calvert formula (Calvert *et al*, 1989), and administered in 250 ml physiological solution, over 30 min. Creatinine clearance was calculated from serum creatinine according to the Cockcroft and Gault formula. Pegylated liposomal doxorubicin was to be administered after carboplatin infusion, in 250 ml of 5% glucose solution, over 1 h.

Criteria for retreatment were: white blood cells ⩾3000 mm^−3^, neutrophils ⩾1500 mm^−3^, platelets ⩾100 000 mm^−3^, absence of organ toxicity ⩾2 (with the exclusion of hair loss). If these minimum conditions were not met, the cycle was postponed by 7 days for a maximum of 2 weeks. If the treatment was delayed for more than 2 weeks, chemotherapy was discontinued due to unacceptable prolonged toxicity. After the first three cycles, in the absence of unacceptable toxicity, patients with objective response or stable disease received further three cycles, for a maximum number of six cycles.

A 20% dose reduction of both carboplatin and PLD was planned in case of grade 4 neutropaenia, if lasting more than 7 days, or platelets <50 000 mm^−3^, if lasting more than 7 days. In case of creatinine clearance <60 ml, the dose of carboplatin was reduced from AUC 5 to AUC 4. In case of palmar–plantar erythrodysesthesia grade 2 or higher, chemotherapy was delayed for up to 2 weeks, until recovery to grade 0–1, and then was applied a 25% dose reduction. If cutaneous toxicity had not recovered after 2 weeks, PLD had to be withdrawn.

No prophylactic use of G-CSF was recommended. In case of grade 4 neutropaenia, even without fever, therapeutic and prophylactic use of G-CSF was allowed.

### Assessment of response

Response was evaluated according to Response evaluation criteria in solid tumours guidelines (Therasse *et al*, 2000). No independent review of the responses was planned. Tumour lesions were categorised as target if they could be accurately measured in at least one dimension as ⩾20 mm with conventional techniques or as ⩾10 mm with spiral CT. All other tumour lesions, including small lesions and truly non-measurable lesions, were categorised as nontarget lesions. Response was assessed every three cycles, with chest X-ray, abdomino-pelvic CT-scan, and other exams, which were positive at baseline evaluation.

### Assessment of toxicity

Toxicity was graded according to National Cancer Institute Common Toxicity Criteria, version 2.0. (National Cancer Institute, 1999). Physical examination and vital signs were recorded before each cycle of chemotherapy. Complete blood counts were performed at baseline and weekly. Laboratory exams (sAST, sALT, total serum proteins, albumin, bilirubinaemia, alkaline phosphatase, lactate dehydrogenase, creatininaemia, blood urea nitrogen, glycaemia, uricaemia, serum electrolytes) were planned at baseline, and then repeated before each cycle and 4 weeks after the end of the last cycle. Left ventricular ejection fraction was assessed by ultrasound before chemotherapy and every three cycles.

### Study design and sample size

The primary end point of the study was the objective response rate (complete response+partial response). A single-stage design for phase II studies has been used to determine the sample size. With *α* error 0.05, power 0.90, minimum acceptable response proportion (p0) 20% and hoped-for response proportion (p1) 40%, 42 patients were needed. At the end of the study, at least 13 objective responses out of the 42 patients treated should be observed, to define the experimental treatment active.

A secondary end point was the description of treatment tolerability. Time to progression, defined as the time from the date of enrolment and the date of the first progression and overall survival, was defined as the time between the date of enrolment and the date of death were also described. Time to progression and overall survival were estimated using the Kaplan–Meier method (Kaplan and Meier, 1958).

## RESULTS

From November 2002 to July 2005, 42 patients were enrolled at 5 Italian Institutions. Main baseline characteristics of the enrolled patients are reported in Table 1. Median age was 64 years (range 31–74). All but one patient had a good ECOG performance status (0 or 1).

Twenty-seven patients had recurrent disease while 15 were advanced. Out of the 27 patients with recurrent disease, 14 had previously received pelvic radiotherapy (50.4 Gy) as adjuvant treatment after surgery. In 6 of these 14 cases also brachitherapy had been given (15 Gy).

Twenty-six patients (62%) completed the planned six cycles. Of the remaining 16 patients, chemotherapy was interrupted before the completion for progressive disease or worsening of disease symptoms (six cases, 14%), for patient's refusal (one patient, 2%), for surgical treatment (four patients, 10%), and for unacceptable toxicity (five patients: four grade 4 haematological toxicity (10%) and one grade 3 heart rhythm). Eleven out of 167 cycles (6.6%) were delayed for 1 week due to toxicity.

Details of worst haematological and non-haematological toxicity are reported in Table 2. One death potentially related to treatment was recorded: death at home, for unknown reasons, after 6th cycle (no previous cardiac history, no examination performed due to sudden death). Grade 3 anaemia was reported in 13 patients (31%), grade 4 anaemia in one patient, with seven patients requiring RBC transfusion. Grade 3/4 neutropaenia was observed in 33 and 14%, respectively, with two patients experiencing febrile neutropaenia. Grade 3/4 thrombocytopaenia occurred in 17 and 5%, respectively, with two patients needing platelet transfusion. No case of bleeding was observed.

Organ toxicity was generally mild: grade 1 heart rhythm two patients (5%), grade 3 heart rhythm one patient (2%), grade 1 renal toxicity two patients (5%), grade 2 liver toxicity one patient (2%). Complete hair loss was reported in two patients (5%). No patient experienced neurotoxicity, and no hypersensitivity reaction was recorded. Palmar–plantar erythrodysesthesia was recorded in 29% of the patients (grade 1 14%, grade 2 10%, grade 3 5%).

In two patients (5%), restaging was not performed, and thus were considered as non-responders according to the intention to treat principle. All the other patients were assessable for response; three complete (7%) and 22 partial responses (52%) were observed, for an overall response rate of 59.5% (95% exact CI: 43.3–74.3). Stable disease was achieved in further 13 patients (31%). According to the study design, the minimum number of response needed to define the treatment active has been obtained.

As on September 2006, 32 disease progressions were recorded. Median progression-free survival was 52.9 weeks (95% CI 44–71.9). With 20 deaths recorded, median survival was 80.1 weeks (95% CI 76.6-NA). (Median follow-up of alive patients was 74 weeks.)

## DISCUSSION

This phase II study shows the feasibility of the combination of carboplatin AUC 5 and PLD, 40 mg m^−2^ in advanced or recurrent endometrial cancer. Promising activity of this combination has been observed.

Treatment of advanced endometrial cancer remains a difficult task for the oncologist due to both the small number of studies performed and the absence of a clear indication of the best therapeutical option to adopt in a population of patients that are often elderly and have associated comorbidities.

Cisplatin–doxorubicin before (Aapro *et al*, 2003; Thigpen *et al*, 2004), and cisplatin–doxorubicin–paclitaxel later (Fleming *et al*, 2004), are the chemotherapy schedules most frequently used worldwide. Particularly, the latter is associated with a toxicity profile that frequently makes it impossible to be given to unselected patients outside a clinical trial (Fleming *et al*, 2004). Furthermore, paclitaxel is not approved for the use in endometrial cancer in most European countries, including Italy. It is commonly believed that there is a need for new schedules of chemotherapy agents that combine efficacy and tolerability, in the attempt to minimise toxicity and maximise quality of life for patients in this setting.

The combination of carboplatin and PLD has been developed in ovarian cancer for platinum-sensitive recurrences (Ferrero *et al*, 2007) and is currently under investigation in first-line (Pignata *et al*, 2006) ovarian cancer, where it has been proven to be safe and effective. The AGO group has recently published their experience with this combination in patients with advanced gynaecologic malignancies, including 19 patients with endometrial cancer, confirming the safe tolerability profile and suggesting a possible advantage in terms of safety compared to the commonly used cisplatin–paclitaxel regimens (Du Bois *et al*, 2006).

Carboplatin and PLD were given to 42 patients in our study. We present here the activity data showing a very interesting response rate of 59.5% (7% complete response) that is at the higher range of activity previously reported for this disease.

These figures were obtained with an acceptable toxicity profile. Toxicity was mainly haematological while the non-haematological toxicity profile was particularly safe. The haematological events were not associated with a significant number of delays in chemotherapy administration and the majority of the cycles were given on time. Neutropaenia and thrombocytopaenia were frequent, with two cases of febrile neutropaenia. No symptomatic thrombocytopaenia was reported. The low rate of neurotoxicity is interesting, given that neurotoxicity is one of the major factors in non-compliance with standard first-line chemotherapy. In addition, the low rate of hair loss could represent a significant advantage compared to the standard regimens of cisplatin, doxorubicin, and paclitaxel.

In this study, liposomal doxorubicin was given every 4 weeks at the dose of 40 mg m^−2^ based on the information present in the literature at the beginning of the study (Verschraegen *et al*, 2001). However, there is now a significant amount of data, at least in ovarian cancer, suggesting that a 3-weekly schedule of carboplatin AUC 5 and liposomal doxorubicin 30 mg m^−2^ is associated with an improved toxicity profile, particularly regarding haematological events, thus suggesting further investigation of this schedule also in patients with endometrial cancer (Pignata *et al*, 2006).

Independently from the dose chosen, 30 or 40 mg m^−2^, and of the schedule, 3- or 4-weekly, we think that the activity of the combination of carboplatin and PLD observed in the present study is at the higher range reported in the literature comparing favourably with platinum-based and paclitaxel-based doublets and triplets (Fleming *et al*, 2004; Thigpen *et al*, 2004). This finding, combined with the safe toxicity profile found in our patients, suggest that a phase III study in order to compare this chemotherapy with the standard cisplatin-based treatment may be recommended.

In conclusion, the combination of carboplatin and PLD shows good activity and favourable toxicity as first-line chemotherapy of patients with advanced endometrial carcinoma, deserving further studies in this setting.

## Figures and Tables

**Table 1:** Baseline characteristics of patients (*n*=42)

	**Median**	**64**	
**Age (years)**	**Range**	**31–74**	**(%)**
Recurrent		27	(64)
Advanced		15	(36)
Stage III		8	
Stage IV		7	
			
ECOG PS	0	28	(67)
	1	13	(31)
	2	1	(2)
			
Grading	1	5	(12)
	2	20	(48)
	3	15	(36)
	NA	2	(4)
			
Histologic type	Endometrioid	35	(83)
	Squamous	1	(2)
	Serous	4	(10)
	Clear cell	2	(5)

NA=not available; PS=performance status.

**Table 2:** Worst toxicity per patient (*n*=42)

	**Worst NCI–CTC grade: number of patients (%)**					
**Toxicity**	**0**	**1**	**2**	**3**	**4**	**5**
Anaemia	8 (19%)	6 (14%)	14 (33%)	13 (31%)	1 (2%)	—
Leukopenia	9 (21%)	7 (17%)	14 (33%)	6 (14%)	6 (14%)	—
Neutropaenia	10 (24%)	5 (10%)	8 (19%)	14 (33%)	6 (14%)	—
Febrile neutropaenia	40 (95%)	—	—	1 (2%)	1 (2%)	—
Neutropenic infection	42 (100%)	—	—	—	—	—
Non-neutropenic infection	41 (98%)	1 (2%)	—	—	—	—
Platelets	22 (52%)	7 (17%)	4 (10%)	7 (17%)	2 (5%)	—
Platelet transfusion	40 (95%)	—	—	2 (5%)	—	—
RBC transfusion	35 (83%)	—	—	7 (17%)	—	—
Allergy	42 (100%)	—	—	—	—	—
Bleeding	42 (100%)	—	—	—	—	—
Fatigue	30 (71%)	7 (17%)	5 (12%)	—	—	—
Heart rhythm	39 (93%)	2 (5%)	—	1 (2%)	—	—
Heart general	42 (100%)	—	—	—	—	—
Pulmonary	42 (100%)	—	—	—	—	—
Fever	42 (100%)	—	—	—	—	—
Weight loss	42 (100%)	—	—	—	—	—
Hair loss	35 (83%)	5 (12%)	2 (5%)	—	—	—
Local reaction	41 (98%)	1 (2%)	—	—	—	—
Skin (including PPE)	30 (71%)	6 (14%)	4 (10%)	2 (5%)	—	—
Anorexia	40 (95%)	2 (5%)	—	—	—	—
Constipation	31 (74%)	7 (17%)	4 (10%)	—	—	—
Diarrhoea	41 (98%)	1 (2%)	—	—	—	—
Nausea	24 (57%)	13 (31%)	5 (12%)	—	—	—
Vomiting	36 (86%)	5 (12%)	1 (2%)	—	—	—
Stomatitis	38 (90%)	1 (2%)	3 (7%)	—	—	—
Liver	41 (98%)	—	1 (2%)	—	—	—
Neuropathy	42 (100%)	—	—	—	—	—
Kidney	40 (95%)	2 (5%)	—	—	—	—
Other	39 (93%)	2 (5%)^*^	—	—	—	1 (2%)^**^

NCI CTC=National Cancer Institute Common Toxicity Criteria; PPE=palmar–plantar erythrodysesthesia; RBC=red blood cell.

^*^Hyperuricemia; ^**^death at home, for unknown reasons, after 6th cycle.
